# GSV: a web-based genome synteny viewer for customized data

**DOI:** 10.1186/1471-2105-12-316

**Published:** 2011-08-02

**Authors:** Kashi V Revanna, Chi-Chen Chiu, Ezekiel Bierschank, Qunfeng Dong

**Affiliations:** 1 Department of Biological Sciences, University of North Texas, Denton, Texas 76203, USA; 2 Department of Computer Science and Engineering, University of North Texas, Denton, Texas 76203, USA

## Abstract

**Background:**

The analysis of genome synteny is a common practice in comparative genomics. With the advent of DNA sequencing technologies, individual biologists can rapidly produce their genomic sequences of interest. Although web-based synteny visualization tools are convenient for biologists to use, none of the existing ones allow biologists to upload their own data for analysis.

**Results:**

We have developed the web-based Genome Synteny Viewer (GSV) that allows users to upload two data files for synteny visualization, the mandatory synteny file for specifying genomic positions of conserved regions and the optional genome annotation file. GSV presents two selected genomes in a single integrated view while still retaining the browsing flexibility necessary for exploring individual genomes. Users can browse and filter for genomic regions of interest, change the color or shape of each annotation track as well as re-order, hide or show the tracks dynamically. Additional features include downloadable images, immediate email notification and tracking of usage history. The entire GSV package is also light-weighted which enables easy local installation.

**Conclusions:**

GSV provides a unique option for biologists to analyze genome synteny by uploading their own data set to a web-based comparative genome browser. A web server hosting GSV is provided at http://cas-bioinfo.cas.unt.edu/gsv, and the software is also freely available for local installations.

## Background

The term 'Synteny' refers to a set of conserved genomic features (*e.g*., genes or other genetic loci) in the same relative ordering on a set of homologous chromosomes. Analysis of genome synteny is particularly important for deciphering a given genome's evolutionary history and identifying its functionally conserved genomic elements [1]. To accomplish this task, biologists often rely on visualization tools for capturing patterns of complicated genomic conservation and rearrangements. Web-based bioinformatics tools are convenient for biologists because users do not need to install and maintain the software. Several web-based synteny visualization tools are currently available, *e,g*., Ensembl SyntenyView [2], NCBI's MapView [3], VISTA [4], SynBrowser [5], GBrowse_syn [6]. However, all these tools only allow users to analyze a small number of pre-selected genome sequences available at those web resources. This limitation is becoming a serious issue since biologists often need to examine synteny for their own sequences of interest that are typically not available at those web resources (*e.g*., genomic sequences produced by local sequencing facility). Although biologists may use certain standalone software to examine their data, the visualization is restricted to the local computers where the software is installed and consequently, the results cannot be easily shared with others without requiring others to install the same software and load the same data set. A better solution would be for biologists to upload their data to a web-based tool allowing their results to be easily shared via an Internet-accessible web site. To achieve this goal, we have developed a web server, Genome Synteny Viewer (GSV), which enables users to upload their own data sets for synteny analysis. Through GSV, synteny can also be visualized along with user-supplied genomic annotation. Besides being hosted as a web server, GSV is also a lightweight package that can be downloaded for free and easily installed elsewhere.

## Implementation

GSV was implemented with freely available open-source software under Linux environment. PHP (http://www.php.net) and MySQL http://www.mysql.com) were used for web interface and back-end database implementation. The PHP:GD library was used for generating dynamic images. JavaScript and jQuery (http://jquery.com) were extensively embedded in the PHP code for interactive browsing features.

The system requires a genome synteny data file and an optional genome annotation data file as inputs (Additional file 1, Figure S1). The format of both files consists of simple tab-delimited columns in plain text. The synteny data file allows users to specify the genomic location of each conserved region, *e.g*., the start and end positions of the conserved regions in each pair of genomes (or chromosomes and genomic segments). Users can also provide additional information such as alignment score, percentage of similarity or identity, etc., to characterize each of the conserved regions. For example, if the BLAST program [7] is used to detect the conserved regions between two genomes, the BLAST E-value or alignment score can be specified in the synteny file to measure the similarity of the regions (Additional file 1, Figure S2). The annotation file is designed for users to list the accompanying genomic features, *e.g*., genes, to be displayed as tracks along the reference genomes. Users can also instruct how each feature is displayed, *i.e*., the shape and color of each annotation track in the annotation file (Additional file 1, Figure S3). Each input file can be submitted either as plain text or in compressed format (*e.g*., .gz or .zip) to facilitate fast file uploads. After input submission, GSV stores the data in a simple relational database (Additional file 1, Figure S4). If the user provides an email address, a notification email (Additional file 1, Figure S5) will be sent with two URL links attached: one to the GSV display page of the current data and the other to access all the previously submitted datasets from the same email address (Additional file 1, Figure S6).

**Figure 1:**
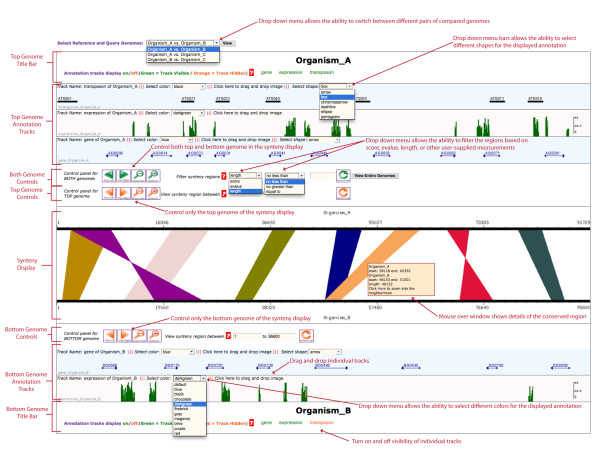
**Overview of GSV Synteny Browser**. In this simulated example, users can switch among three pairs of genomic sequences for analysis (*i.e*., *Organism A vs. Organism B*; *Organism A vs. Organism C*; *Organism B vs. Organism C*). The synteny view of *Organism A vs. Organism B *is displayed at the center of the browser. Each selected genomic sequence is represented as a black horizontal ruler. Users can *zoom in/out, pan left/right *or select specific genomic regions for a customized display. Users also have the choice to display the two entire genomic sequences by clicking the button *View Entire Genomes*. In this example, the conserved genomic regions between *Organism A *and *Organism B *are shown in colored blocks. Above the synteny view, three annotation tracks are displayed for *Organism A *(*i.e*., the "gene" track in blue arrows representing the predicated genes for each genome, the "expression" track in green bars representing whole genome tiling array hybridization intensity, and the "transposon" track in black boxes representing putative repetitive elements). Below the synteny view, only the "gene" and "expression" tracks are displayed for *Organism B*. Note that the "transposon" track is turned off for *Organism B*, but can be turned on through *Annotation tracks display on/off *from the control panel. Additional control panel at bottom of the display (not shown here, see Additional file 1, Figure S7) allows users to export their customized figures in the *png *format. See text for additional description of the browser's functionality.

A genome synteny browser is implemented to display the user-supplied data (Figure 1). The unique feature of the GSV synteny browser is that it presents two selected genomes in a single integrated view while still retaining the browsing flexibility for individual genomes. At the top of the display, a pull-down menu is available that allows users to select the desired pair of genomes for visualization. If multiple genomes are presented in the user-supplied synteny data, this pull-down menu allows users to easily switch among different pairs of genomes for analysis. The genome synteny view is presented at the center of the display. Each selected genome is represented as a black horizontal ruler with tick marks showing its genomic position. Conserved regions between the two selected genomes can be either displayed as colored lines or blocks upon the user's choices. On mouse-over of a displayed conserved region, a pop-up menu is displayed for its precise boundary as well as a zoom function centred on this particular region. Users can *zoom in/out, pan left/right *or select specific regions on both or individual genomes for display by using the embedded control panels. Users can also *filter *the conserved regions based on their associated *characteristics *as shown in the filter pull-down menu. By default, users can filter the conserved regions by length. If additional characteristic measurements are provided in the synteny data files (*e.g*., alignment score, E-value), they will also appear as filter options in the pull-down menu. Selecting, or filtering, allows users to focus on the regions of interest that meet certain criteria (*e.g*., by applying stringent BLAST E-value to only display highly conserved regions).

If an annotation file is also provided, genomic tracks will be displayed for each selected genome. The track display is similar to other standard genome browsers with the following novel functions. Users can dynamically change the color or shape of the selected tracks on the fly. For example, users can change the display of genes from red boxes to blue arrows simply by using the pull-down menus *Select color *and *Select shape *available at each track display. Users can also re-order the displays of different annotation tracks by dragging the selected tracks to different positions (*e.g*, placing a track of predicted genes on top of an expression track to display potential active genes). An additional control panel allows users to hide or show each annotation track. Besides browsing the data, users can also export customized figures (either the entire figure or part of it) as Portable Network Graphics (png) image files for publications or presentations (Additional file 1, Figure S4).

## Results and discussion

Enabled by next-generation DNA sequencing technologies, individual biologists can sequence a large variety of species, strains or specific genomic regions of interest. However, centralized web databases often do not have the resources for displaying such highly individualized data set to satisfy every user's specific needs. GSV provides a unique option for biologists to upload their own data set to a web-based synteny browser for analysis. Its intuitive web interface allows users to easily examine conserved genomic regions in the context of the accompanying genome annotations. The web-accessible results can be easily shared with the research community (*e.g*., collaborators in other institutions, or supplementary materials for journal publication), which is an important feature that standalone tools do not normally have.

GSV users need to prepare two data files, the mandatory synteny file for specifying genomic positions of conserved regions and the optional annotation file for listing the annotated genomic features. The synteny data file has an "open-ended" format that allows users to provide flexible numerical measurements on each conserved genomic regions. For example, some users may wish to use alignment scores to measure how conserved each region is, but others might choose the percentage of similarity, BLAST E-values, etc. Such alignment scores, similarity scores, E-values, or any other numerical measurements can be used as additional columns in the synteny file. The annotation file format allows users to also specify the color and shape for the display of each genomic feature in addition to their genomic locations. If necessary, users can even configure the display of a single genomic feature, *e.g*., highlighting a particular gene of interest in a different color than the rest of genes in the same gene track. The new GSV formats were developed because none of the existing formats can achieve the above goals easily (the GSV formats may also be modified based on users' feedback in the future). Anyone who has basic programming skills, *e.g*., collaborators in the local bioinformatics center or computer science department, can easily help biologists convert any raw outputs produced by other programs into the GSV formats. Sample Perl scripts for converting BLAST output, BLASTZ [8] output, and GFF3 (http://gmod.org/wiki/GFF3#GFF3) format data files into the appropriate GSV formats are provided in the GSV package.

Although the GSV web server is open to the entire research community to use, the entire package can also be easily installed on different servers, *e.g*., local genomics facilities. Unlike the installation of other sophisticated software packages, the installation of GSV is straightforward and all of the pre-requisites are already part of the standard Linux distribution (it takes about 30 minutes to install the entire package).

## Future work

Users may provide the data for multiple pairs of genomes in the synteny file, however only two genomes can be displayed for comparison at any given time in the current version of GSV. Developing intuitive web interfaces for simultaneously displaying synteny among multiple genomes and their associated genome annotation is still a very challenging task. We are currently experimenting with some designs to upgrade GSV for accommodating multiple genomes in an integrated display. Once fully implemented, the new release of GSV will be published in a separate paper.

## Conclusions

A major challenge for bioinformaticians is to develop suitable computational tools that assist biologists in analyzing diverse data sets. The current existing genome synteny web servers are not always flexible enough to accommodate the visualization needs from individual users. To our best knowledge, GSV is the only web-based synteny visualization tool that allows biologists to upload their own data set. Its light-weighted architecture also allows others to easily install in their local servers.

## Availability and requirements

The GSV web server is accessible at (http://cas-bioinfo.cas.unt.edu/gsv) and the open-sourced software is also available from the web site for local installation under the terms of the GNU General Public License (http://www.gnu.org/licenses/gpl.html). GSV is portable across Linux distributions, and compatible with PHP 5.2.6 (or higher version) and MySQL 5.0 (or higher version). GSV installation has been tested on Debian Lenny and Ubuntu Lynx. GSV can be viewed with FireFox 3.6.15, Safari 3.0, Internet Explorer 7.0 and Chrome 11.0. The GSV website will be updated to contain the latest information on operating systems and software compatibility.

## Competing interests

The authors declare that they have no competing interests.

## Authors' contributions

Both KR and CC designed and implemented the software. EB contributed to web interface design. QD conceived the project and prepared the manuscript. All authors read and approved the final manuscript.

## Supplementary Material

Additional file 1 **A Microsoft Word document for additional supplementary figures to illustrate the GSV system and features**. Click here for file
