# Supplementary material for: GSV: a web-based genome synteny viewer for customized data
Source: BMC Bioinformatics. 2011 Aug 2;12:316. doi: 10.1186/1471-2105-12-316 (PMC3199762; doi:10.1186/1471-2105-12-316)

**Supplementary f**igure 1 legend. **GSV home page.** At http://cas-bioinfo.unt.edu/gsv, users can upload their synteny file and optional annotation file. Users also have the option to supply their email addresses in order to receive results via email (Additional file 1, Figure S5). Example data files are provided at this page for first-time users to explore the functions of GSV.

**Supplementary f**igure 2 legend. **Sample synteny file.** The synteny file is a tab-delimited plain text file. The first six columns are mandatory (shown in green background) for describing the genomic coordinates of each of the conserved regions in two genomic sequences. Column #1 is the ID of the first genomic sequence for comparison; Column #2 is the start coordinate of the conserved region on the first genomic sequence; Column #3 is the end coordinate of the conserved region on the first genomic sequence; Column #4 is the ID of the second genomic sequence for comparison; Column #5 is the start coordinate of the conserved region on the second genomic sequence; Column #6 is the end coordinate of the conserved region on the second genomic sequence. An inversion event can be specified as having a larger start coordinate than end coordinate. Additional columns are optional (shown in pink background) for providing the numerical measurements of the conserved genome regions. In this example, column #7 is the alignment score and column #8 is the BLAST E-value for measuring the similarity of the regions. Users may provide their own additional columns (the number of such additional columns are not restricted, but the aforementioned columns must be numerical types and the names of the columns should not contain any space characters). Note that data for the multiple pairs of genomic sequences can be stored in one single file, *e.g.*, Organism A *vs.* Organism B, and Organism A *vs.* Organism C in the figure. GSV only displays linear genomic sequences. Thus, the genomic coordinates of circular genomes should be specified as if they were linear (*e.g.*, using the replication origin for bacteria genome as position 1), which is a common practice used by other standard genome browsers.

**Supplementary f**igure 3 legend. **Sample annotation file** The annotation file is a 9-column tab-delimited text file: Column #1: ID of the reference genome; Column #2: Start position of the genomic feature; Column #3: End position of the genomic feature; Column #4: Strand orientation of the genomic feature, i.e., "+" for sense strand and "-" for the reverse complementary anti-sense strand; Column #5: Feature name, e.g., ID of a particular gene; Column #6: Feature value. This column is typically applied to features that are associated with some numerical values, e.g., microarray hybridization intensity for measuring gene expression level, which can be used for XY plot; Column #7: Track name. All of the common features are displayed as a horizontal track along the reference genome. For example, all the predicated genes can be displayed in a track named as “gene”; Column #8: Track shape. Users can choose from a list of predefined shapes, e.g., arrow, dashline, xy-plot, pentagram, Christmas arrow, box, and ellipse, to display their features for the selected tracks. Column #9: Track color. Similarly, users can choose from a list of colors for the feature display, e.g., red, blue, and green. Note that data for multiple genomes can be stored in one single file. Column #4, #5 and #6, may contain a dot if data is neither available nor applicable. Note that annotations for multiple genomes can be combined in one file as shown in this figure for the *Organism A, B,* and *C.*

**Supplementary figure 4 legend**. **Overview of the GSV database schema.** The GSV backend database consists of one “userinfo” table and potentially a series of “synteny” and “annotation” tables. The “userinfo” table is created when GSV is installed. Its purpose is to link the user-supplied email addresses to all the records that the same users have submitted to the server. In the “userinfo” table, its "id" column is the auto-increment primary key in the userinfo table. The column "hash" is MD5-encrypted users' email addresses for tracking the users’ uploading histories. The columns “synfilename” and “annfilename” store the names of user-uploaded synteny file and the annotation file, respectively. The column "url" refers to the web address needed to access users’ results, which is a concatenated string of the encrypted email address and session_id. Neither the “synteny” table nor the “annotation” table are created until users upload their data set. For each user-supplied data, a new synteny and annotation table is created. A “session_id” is appended to the table name to differentiate different users' data table. The “session” id is generated by combining the current system timestamps and process ID. The basic “synteny” and “annotation” table structures are described in the Additional file 1, Figure S2 and Additional file 1, figure S3, respectively. Note that the columns “score” and “email” in the “synteny” table represent examples of any additional numerical columns for measuring the extent of genomic region conservation as discussed in the main text. The “length” column in the “synteny” table stores the length of each conserved genomic region, dynamically computed as the smaller value of *|genome_1_end_coordinate – genome_1_start_coordinate| + 1* and *|genome_2_end_coordinate – genome_2_start_coordinate| + 1*. Additional details on the GSV architecture can be found in the “ARCHITECTURE” file in the GSV downloadable package.

**Supplementary figure 5 legend. Email notification of user’s result.** If a user’s email address is submitted, the user will be notified through an email which contains two URL links, one to the GSV display page of the current data and the other to access all the previously submitted datasets from the same user.

**Supplementary figure 6 legend. History page.**  A history page is maintained for accessing all the previously submitted datasets from the same email address and can be accessed through the link provided in the email sent by GSV. In order for the history page to maintain a record of the users information, a email must be submitted each time GSV is used.

**Supplementary figure 7 legend. Image export control panel.** At the bottom of the GSV display (fig. 1), an image control panel allows users to generate their customized figures (*e.g.*, the entire image or part of the image with selected tracks) and download in the png format. The image is fully customizable in that each track can be included or excluded. Once the user clicks the “create Image” button, the customized image will open in a new window, where the users can then download the image.

**Supplementary f**igure 1

**
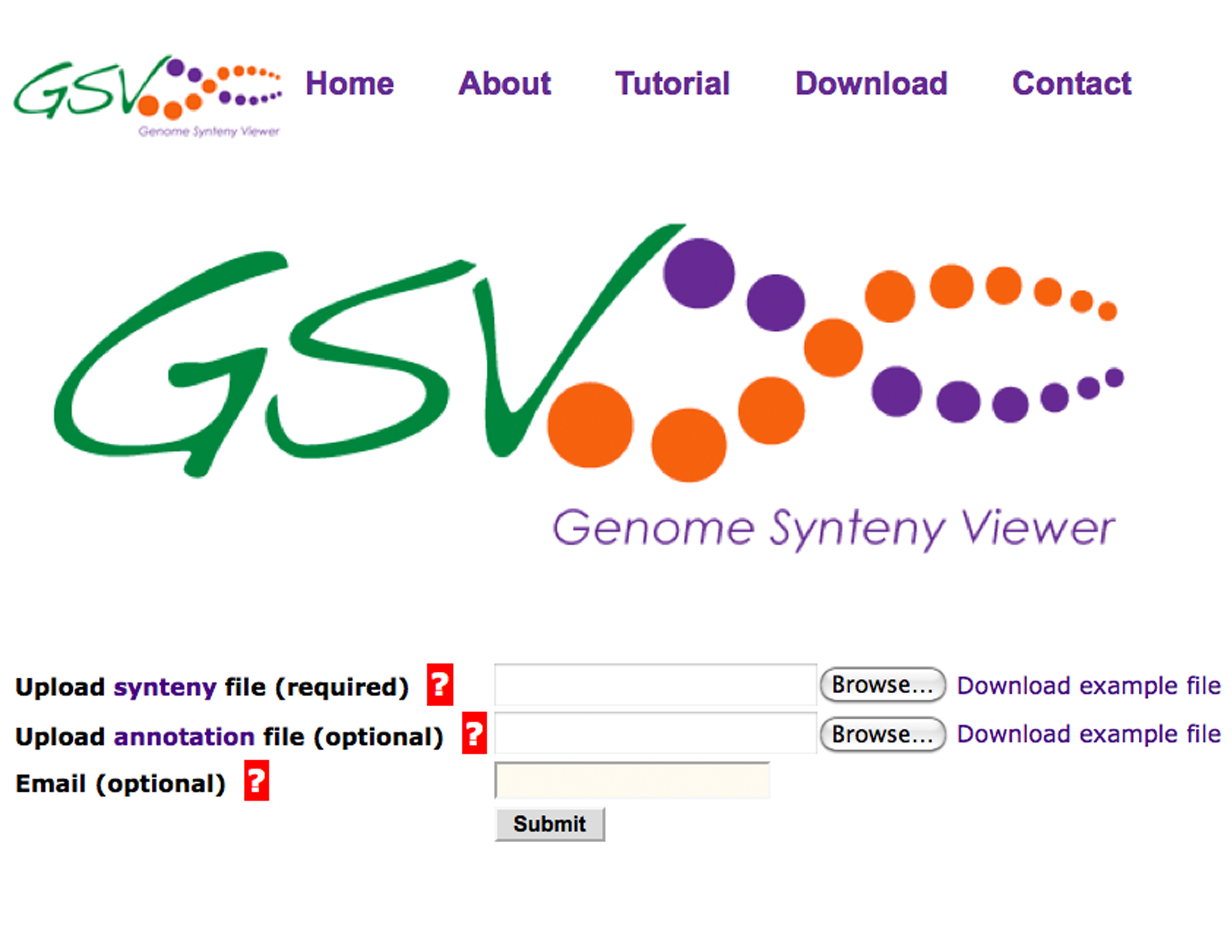
**

**Supplementary f**igure 2


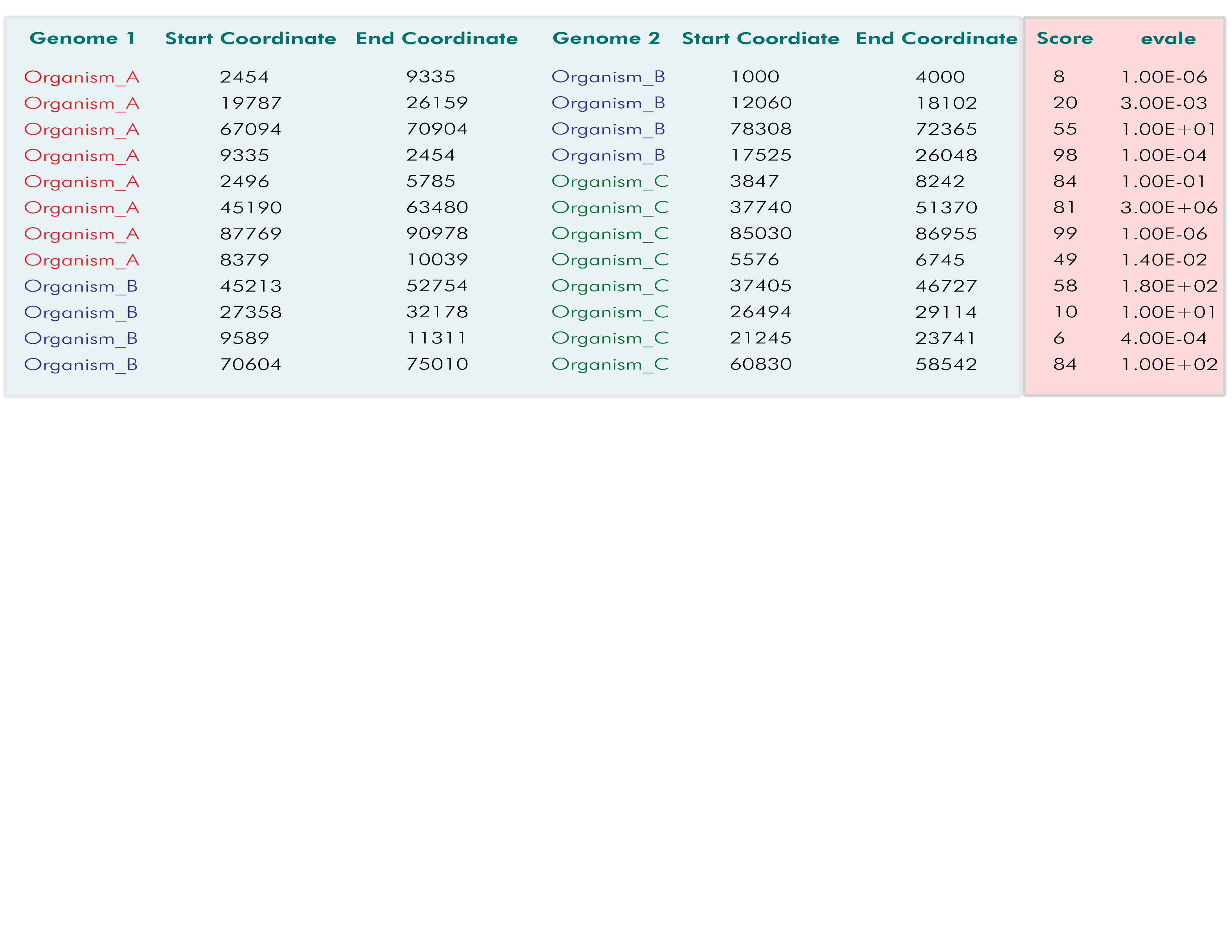


**Supplementary figure 3**

**
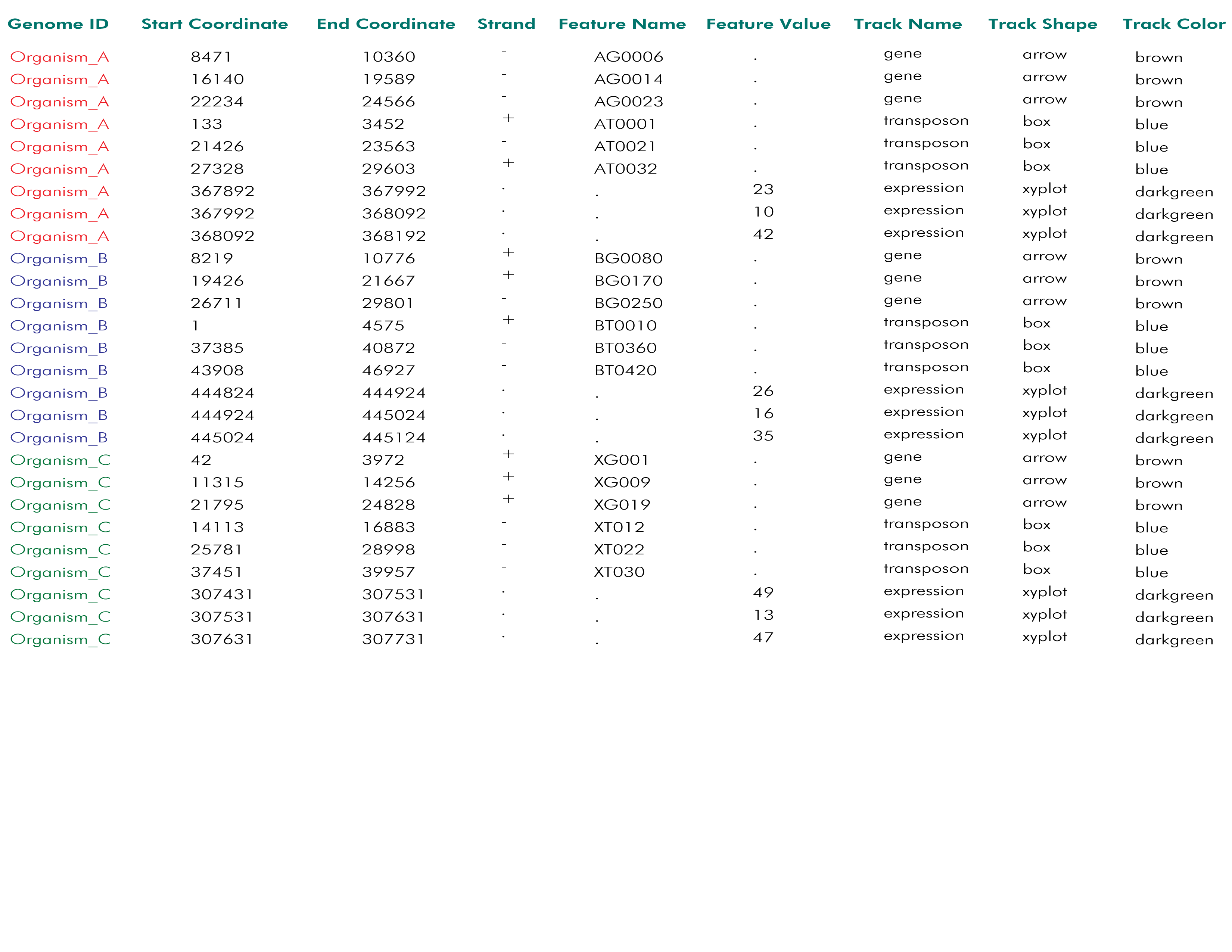
**

**Supplementary figure 4**


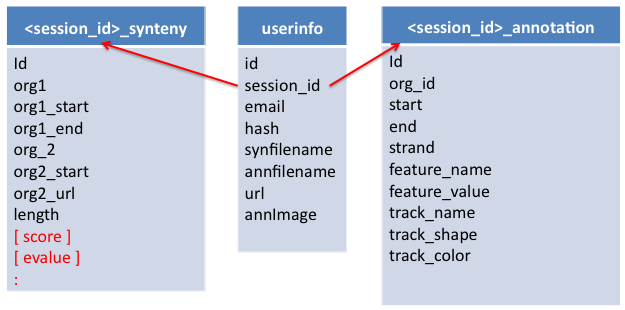


**Supplementary figure 5**

**
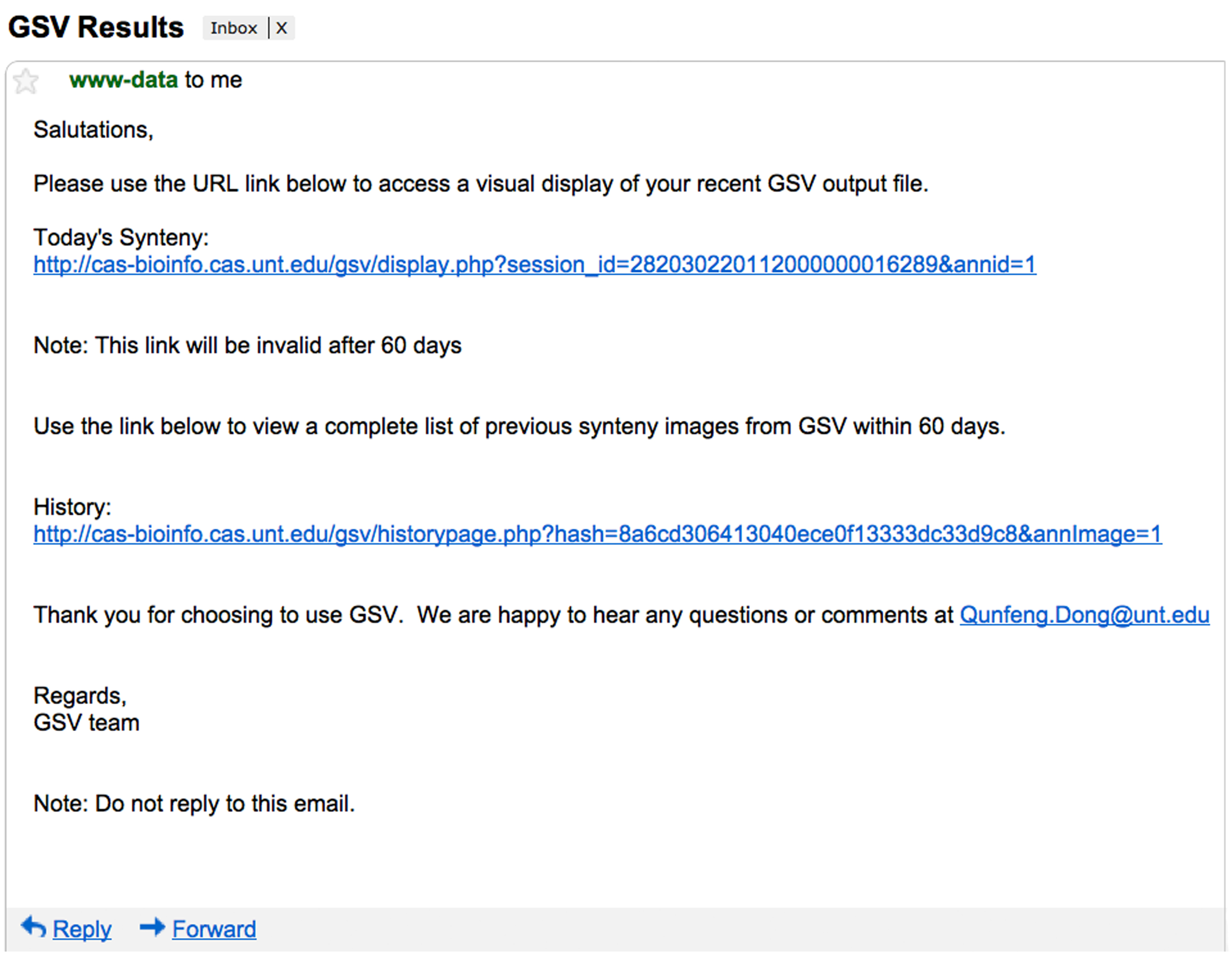
**

**Supplementary figure 6**

**
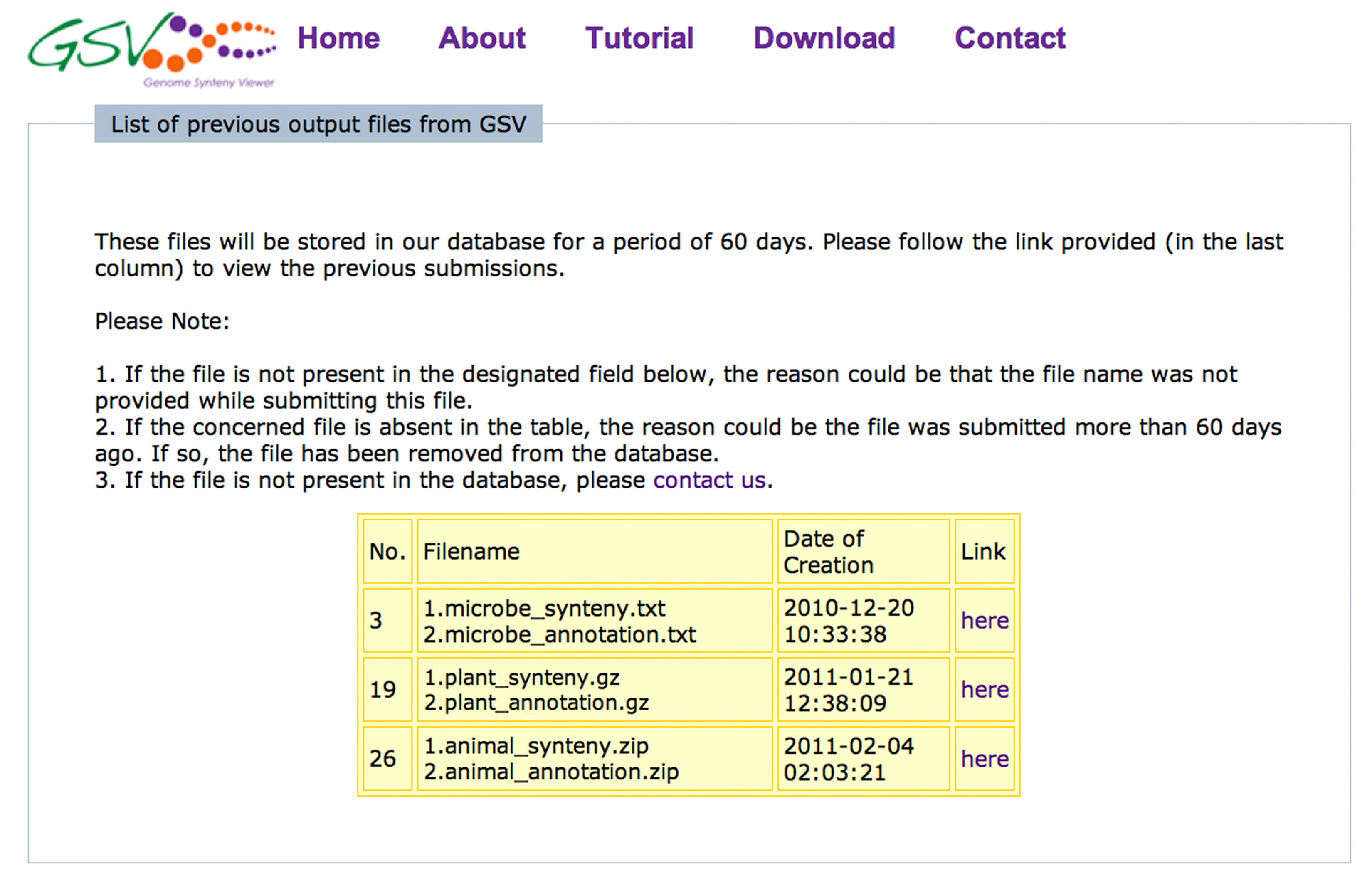
**

**Supplementary figure 7**


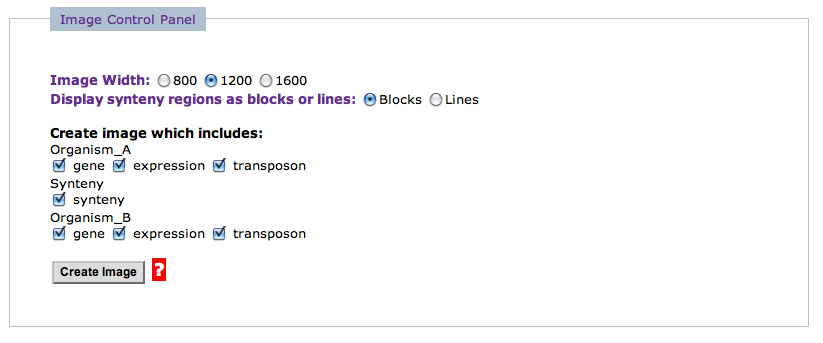

Supplement: Additional file 1 — A Microsoft Word document for additional supplementary figures to illustrate the GSV system and features. [file 1471-2105-12-316-S1.DOC]
